# Sexy Faces in a Male Paper Wasp

**DOI:** 10.1371/journal.pone.0098172

**Published:** 2014-05-21

**Authors:** André Rodrigues de Souza, Carlos Alberto Mourão Júnior, Fabio Santos do Nascimento, José Lino-Neto

**Affiliations:** 1 Departamento de Entomologia, Universidade Federal de Viçosa, Viçosa, Minas Gerais, Brasil; 2 Departamento de Fisiologia, Universidade Federal de Juiz de Fora, Juiz de fora, Minas Gerais, Brasil; 3 Departamento de Biologia, Faculdade de Filosofia Ciências e Letras de Ribeirão Preto da Universidade de São Paulo, Ribeirão Preto, São Paulo, Brasil; 4 Departamento de Biologia Geral, Universidade Federal de Viçosa, Viçosa, Minas Gerais, Brasil; Arizona State University, United States of America

## Abstract

Sexually selected signals are common in many animals, though little reported in social insects. We investigated the occurrence of male visual signals mediating the dominance relationships among males and female choice of sexual partner in the paper wasp *Polistes simillimus*. Males have three conspicuous, variable and sexually dimorphic traits: black pigmentation on the head, a pair of yellow abdominal spots and body size differences. By conducting behavioral assays, we found that none of the three visual traits are associated with male-male dominance relationship. However, males with higher proportion of black facial pigmentation and bigger yellow abdominal spots are more likely chosen as sexual partners. Also, after experimentally manipulating the proportion of black pigment on males' face, we found that females may evaluate male facial coloration during the choice of a sexual partner. Thus, the black pigmentation on *P. simillimus* male's head appears to play a role as a sexually selected visual signal. We suggest that sexual selection is a common force in *Polistes* and we highlight the importance of this group as a model for the study of visual communication in insects.

## Introduction

Males often present conspicuous traits that act as signals of quality mediating competition for reproductive opportunities [1], [2]. Thus, nests, exaggerated plumage, bright colors, horns, acoustic and behavioral repertoires are commonly used to convey information about an individual's ability, such as foraging, fighting, resistance to parasites and good genes [3]–[13]. These sexually selected signals drive the disputes between males for access to females (intrasexual competition), the female choice of sexual partners (intersexual competition), or both.

Males of many eusocial Hymenoptera are potentially subjected to a high sexual selection pressure [14]. For example, in many species, the mating system is based on leks, where there is a high bias in reproductive success among males [15]–[19]. In addition, females are typically monogamous [20] and, consequently, the choice of sexual partner may be important. Thus, sexually selected signals are expected to mediate the competition for reproductive opportunities. However, there is a lack of data concerning sexually selected traits in social insects and some researchers have claimed that sexual selection in these insects is weak [21].

To date, there is only one example of sexually selected signal in eusocial insects. Males of *Polistes dominula* have a pair of yellow spots on their abdomen that act as visual signals used to mediate both the dominance relationships among males and female choice of sexual partners [22]. Males of this species form leks [16], [18], in which they aggressively compete for dominance positions. Females visit several leks and evaluate males before mating [18]. Additional research on the occurrence of sexually selected signals in *Polistes* is motivated because its body coloration is striking, highly variable and sexually dimorphic [23]. Further, in females, visual cues have been used to signal individual identity [24] and individual quality [25], [26], [27] and thus, the study of paper wasp coloration is a promising avenue for understanding visual communication in insects.

We investigated the occurrence of sexually selected signals in males of the social wasp *Polistes simillimus*. First, we identified three conspicuous, variable and sexually dimorphic male visual traits: black pigmentation on the head, a pair of yellow abdominal spots and body size. Then, we performed behavioral essays in order to test whether each of these traits are associated with dominance relationships among males and/or female choice of sexual partner. Further, we also experimentally manipulated the black pigmentation on the head to test whether females evaluate this male coloration.

## Materials and Methods

### Ethic Statement

The collection of colonies and the performed behavioral experiments comply with the current laws in Brazil. No specific permits are required for collection of wasps, and the species used in the experiments is not endangered or protected in Brazil.

### Wasp Collection and Maintenance

A total of 15 male-producing colonies of *P. simillimus* were collected in Viçosa, Minas Gerais state, Southeastern of Brazil (20° 48' S, 42° 51' W, elevation 800 m), between January and April 2013. In the laboratory, the nests were kept in plastic containers of 1 L. Adults emerged in the field (i. e. adults already emerged at the time of colony collection) were frozen. Adults emerged from nests after collection were individualized in plastic containers of 0.5 L and fed with honey *ad libitum*. Virgin males between 7–22 days and virgin females between 10–25 days after emergence were used in behavioral assays and subsequently frozen. Wasps used in a given experimental trial came from locations at least 1.5 km from each other. Each wasp participated in a single behavioral test.

### Sexual Dimorphism

We analyzed sexual dimorphism by using adult males and females collected in the field, as well as new adults emerged in the laboratory. Before analyzes, wasps remained frozen at −20°C for about 2–10 weeks. Freezing for this period does not change the shape or color of wasps. We searched for sexual dimorphism in the proportion of black pigment on the head, the morphology of abdominal yellow spots and the head size. These parameters were measured as follow: The head and abdomen of each individual were previously separated from the rest of the body. Images were captured with a digital camera (Canon A-620) attached to a stereomicroscope (Stemi 2000-C). We positioned heads in frontal view and in frontal view slightly inclined downward, and the abdomen in the right and left lateral views [22]. We then analyzed the images by using Image Pro Plus 5.0 application.

**Figure 1:**
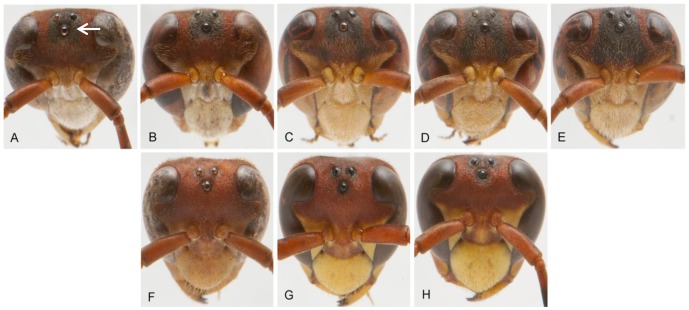
Variation in the proportion of black pigment in heads (arrow) of males (A–E) and females (F–H). Note that males have larger and more variable spots than females.

The black pigmentation on male's face is concentrated in the upper portion of the head, around the ocelli (Fig. 1). It may extend vertically from the line immediately below the antennal sockets to the line above the upper limit of the eyes. Laterally, the black pigmentation may extend to the inner edge of the eye. By using images of the head slightly inclined, we calculated the proportion of black pigmentation in this region, excluding the area of the ocelli and antennal sockets (Fig. S1).

The pair of abdominal spots was evaluated by using the spot shape index, or SI [22]. The index considers the width and height of the largest ellipse that can be inserted into the spots as well as the total area of the spots (Fig. S2). Thus, SI = A/(Pi×(1/2L)×(1/2H)), where width (W), height (H) and the area (A) corresponds to the mean values obtained for the right and left spot of a given wasp.

For each wasp, we measured the maximum head width in front view (Fig. S3), as this measure is a strong predictor of body size [28].

### Association between Male Visual Traits with Dominance and Mating Success

To investigate which traits are associated with male-male dominance and/or female's choice of sexual partner, we conducted (i) dominance trials, in which two unfamiliar males were introduced to each other and (ii) mating trials in which two unrelated males were simultaneously presented to an unrelated female. Both kinds of trials took place in a glass arena (8×8×2 cm) where wasps were allowed to interact during one hour. As there were always two males in each trial, one of them received a small amount of white paint on the dorsal part of the thorax, allowing individual identification. This experimental design is similar to that used to study *P. dominula* males' sexual signals [22].

### Experimental Manipulation of Black Pigmentation on the Male Face

**Figure 2:**
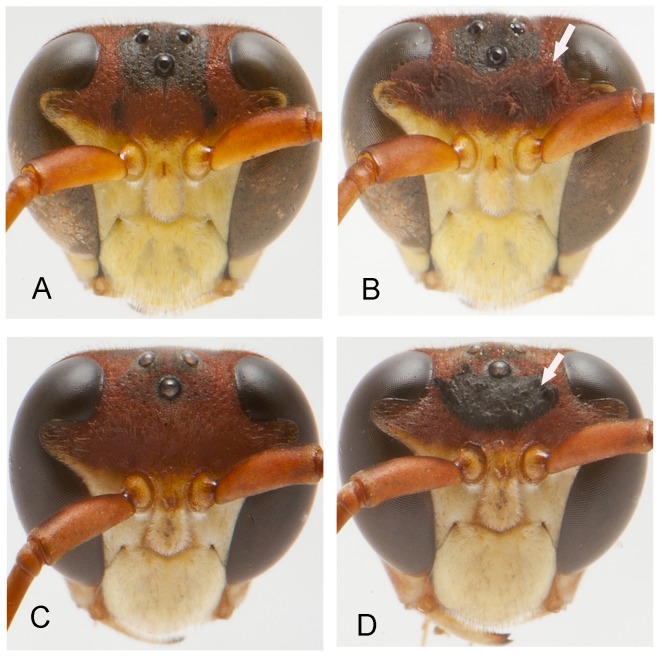
Experimental paint manipulation of males' black pigmentation on the head. Males were manipulated to have low (A, before; B, after manipulation) or high black pigmentation (C, before; D, after manipulation). The arrows indicate the manipulated area.

We tested whether females evaluate the black pigmentation on the male face during the choice of a sexual partner. To do so, the same kind of mating trial was performed (one female and two males), but this time we manipulated the proportion of black pigmentation on the male's face by using brown and black paints (Acrilex®). One male in each of these trials had a portion of his natural black pigmentation on the face covered with brown ink, and to control, he received a similar amount of black ink on a naturally black area. In the other male of the pair, black facial pigment was increased by the addition of black ink on a naturally brown portion of the head, and to control, a similar amount of brown ink was added on the original brown area. As a result, each male of a given pair had a greater or lesser proportion of black pigment in the face (Fig. 2), though both received a similar amount of brown and black inks. Wasps were painted one day before being used in the behavioral experiments. This period is sufficient for individuals to become familiar to the presence of ink on their body [29]. The experimental manipulation of ornaments to observe the response of the receptor has been frequently used to test whether a trait function as a sexual signal [30]–[33].

### Behavioral Analysis

A single observer, blind to the males black pigmentation on the head, the morphology of abdominal spots and the body size, directly recorded all interactions between wasps. Aggressive behaviors such as biting, darting and mounting are common among *Polistes* males [17], [34], [35] and are used to infer their dominance relationships [22]. In each dominance trial, the observer recorded the direction and frequency of aggression between males. We categorized the most aggressive male as dominant and the less aggressive as subordinate.

*Polistes* females are generally larger and more aggressive than males. Thus, it is expected that they are able to accept or reject mating [15]. In each sexual trial, the observer recorded the frequency of mating between female and each of the two males. We confirmed insemination by the presence of sperm in the spermatheca of the female a day after the behavioral test. Thus, we classified males that inseminated females as chosen and males who did not inseminated females as not chosen.

### Statistical Analyzes

We studied sexual dimorphism on visual traits by using General linear models (GLM). First, we run a GLM to see how the proportion of vertex black pigmented is influenced by sex and body size; we also tested whether the spot shape index is influenced by sex and body size; finally, we run a GLM to verify how body size is influenced by sex.

To test whether male visual traits were associated with male-male dominance or female mate choice, we performed two separated generalized mixed effects models (GLZ) for a binomial response variable. In one of the models, whether or not a male was dominant was input as the dependent variable, while body size, proportion of vertex black pigmented and spot shape index were entered into the analysis as the independent variables and finally, males' age, painting on the thorax and nest origin were included as random effects. The other model was similar, but the dependent variable was whether or not a male copulated instead of whether or not a male was dominant.

We used the non parametric bilateral Fisher's exact test to verify if males experimentally manipulated to have a high or a low proportion of black pigment on the head differed in the probability of being chosen by the female as sexual partners, The same test was used to verify the effect of marking with white paint on the probability of a male being dominant and/or being chosen as the female sexual mate.

Descriptive and inferential analyzes were performed by applications SPSS 15.0 and Statistica 12.0. The level of significance was set at 5%.

## Results

### Sexual dimorphism

**Figure 3:**
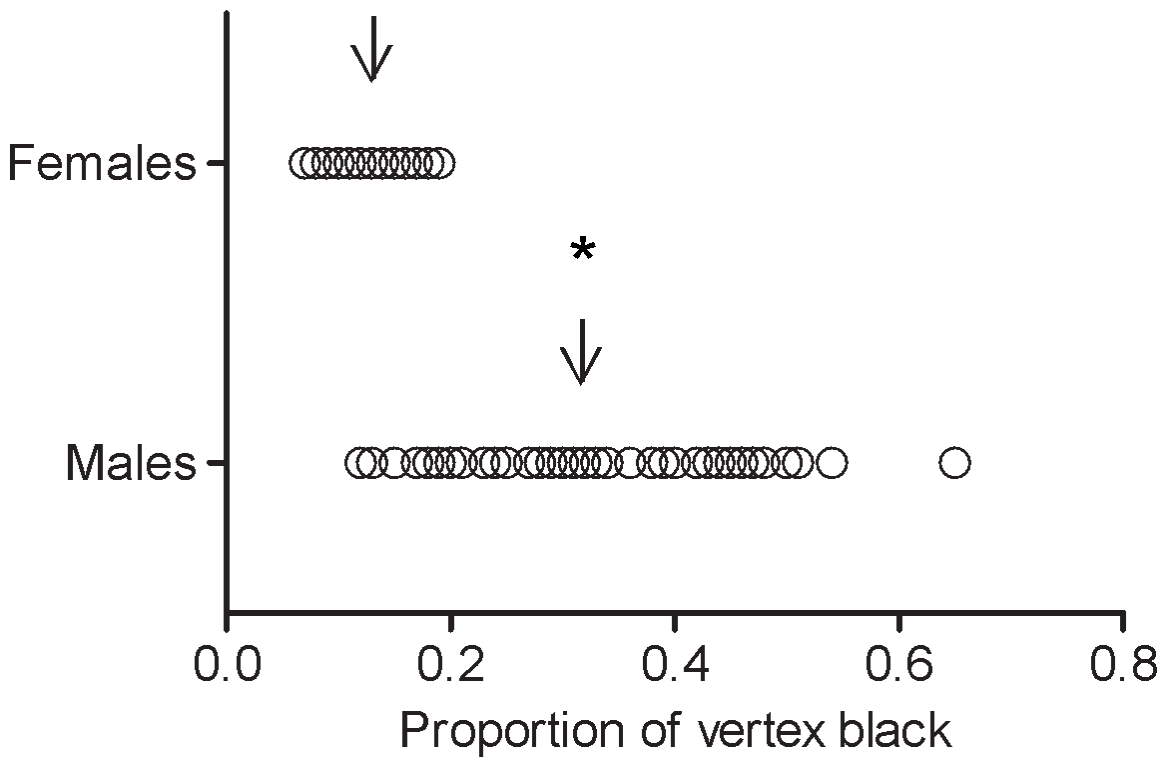
Variation in the proportion of black pigment in heads of males and females. The proportion of black pigmentation on the head is higher and more variable in males than in females. Each circle represents one individual and the arrows indicate the mean. * Indicates statistical difference between the classes (see text).

**Figure 4:**
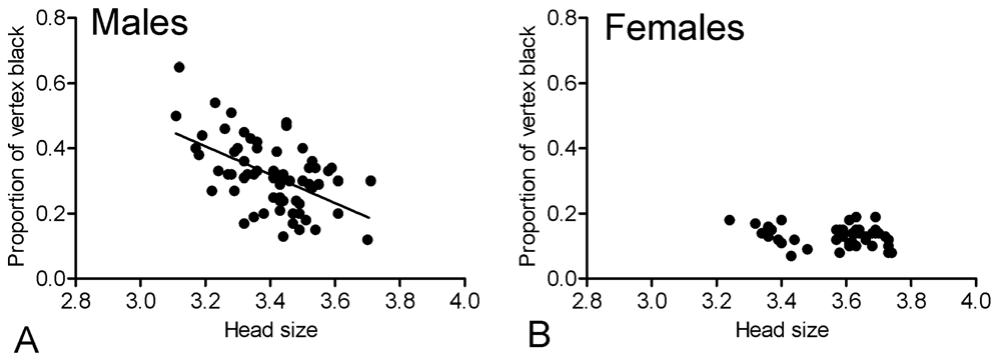
Relation between head size and proportion of vertex black pigmented. Males have a stronger negative relation compared to females.

The proportion of black pigmentation on the head was influenced by sex (F_1,109_ = 14.7991, P = 0.0002, N = 113) so that males' black pigmentation is higher and more variable than in females (males  = 31±10% (range: 12–64%), females  = 13±3% (range: 7–19%), Fig. 3). Also, the black pigmentation was influenced by body size (F_1,109_ = 22.3330, P<0.0001, N = 113) and the interaction among body size and sex (F _1,109_ = 12.0893, P = 0.0007, N = 113). It means that body size alone affects the black pigmentation on the head but this effect is different in males and females. Specifically, the proportion of black facial pigment is strongly negatively related with body size of males, but this relation seems to be weak in females (Fig. 4).

The SI was influenced by sex (F_1,109_ = 17.3797, P<0.0001, N = 113), so that males had larger and more irregular abdominal spots than females (SI males abdominal spots: 0.0138±0.0032 (range: 0.0098–0.0244), females: 0.0121±0.0014 (range: 0.0092–0.0156), N = 113; Figs. S4 and S5). Also, the SI was influenced by body size (F_1,109_ = 17.3277, P<0.0001, N = 113) and the interaction among body size and sex (F_1,109_ = 17.1664, P<0.0001, N = 113). It means that body size alone affects SI but this effect is different in males and females. Specifically, the SI is strongly negatively related with body size of males, but this relation seems to be weak in females (Fig. S6).

Finally, body size is affected by sex (F_1,109_ = 45.32, P<0.0001, N = 113), so that males are smaller than females (males head size  = 3.4±0.13 (range: 3.11–3.71), females head size  = 3.58±0.13 (range: 3.24–3.74), Fig. S7).

### Association between sexually dimorphic traits with male dominance and female choice

Aggressive behaviors were observed in 16 of 39 trials with unmanipulated males. In each trial, aggression between males was low and always unidirectional. Dominant males exhibited 0–5 darts and 0–3 bites on subordinate males. Dominance relationship among males was not associated with the proportion of black pigment on the head (χ^2^ = 0.2627, P = 0.6082, N = 16), the irregularity of the abdominal spots (χ^2^ = 0.1390, P = 0.7091, N = 16) or the head size (χ^2^ = 0.2786, P = 0.5975, N = 16).

**Figure 5:**
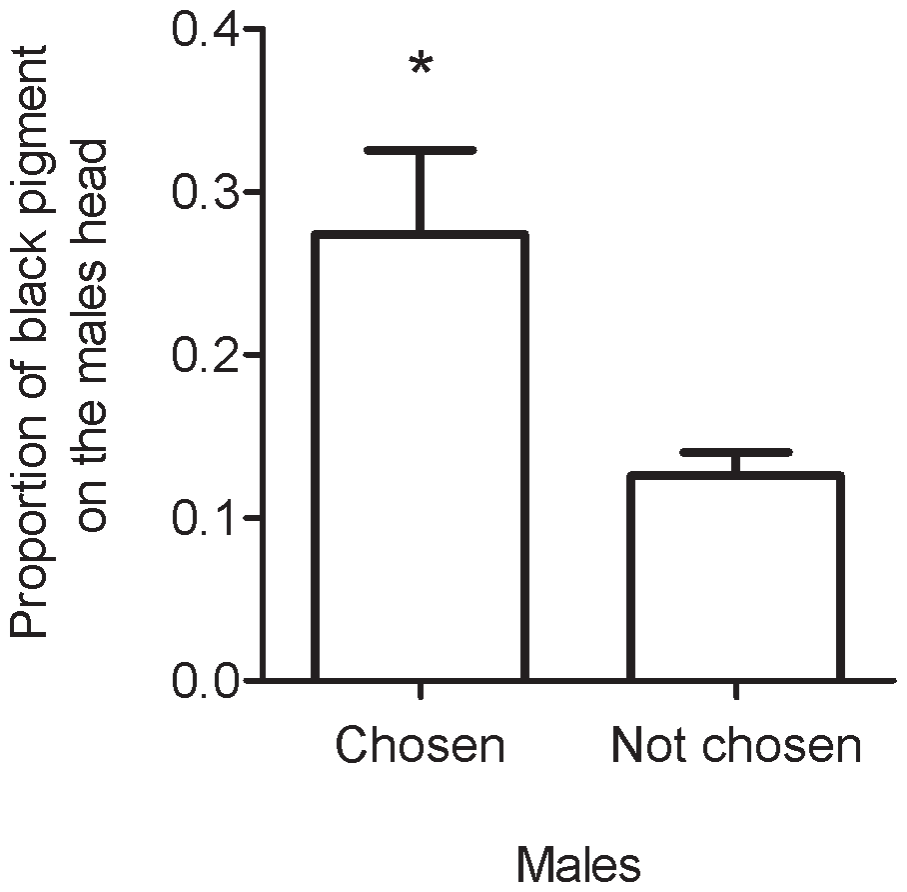
Proportion of black pigment of males chosen and not chosen by females as sexual partners. Mean and standard deviation are presented. * Indicates statistical difference between the classes (see text).

During the unmanipulated mating trials, aggressive interactions between males were not observed. Copulations occurred in 10 out of 44 trials. Females rejected males in different ways: when males tried to mount, females flew to another part of the arena or bite and/or stung males, yet some males were able to mount females. In this case, females moved the abdomen, avoiding genital contact. Alternatively, females accepted copulation by allowing genital interlocking. In each sexual trial, we observed 0–4 copulations, each lasting 6–20 s. Females copulated with only one of two males available in each trial. Copulations were always associated with sperm transfer as verified by checking the spermatheca. The female choice of sexual partner was associated with the male proportion of black pigment on the head (χ^2^ = 9.4800, P = 0.0020, N = 10), so that chosen males have bigger black spots than non chosen ones (Fig. 5). Female choice of sexual partner was also associated with male SI (χ^2^ = 8.1154, p = 0.0043, N = 10), so that chosen males have bigger yellow abdominal spots than non chosen ones (Fig S8.), and with the interaction between SI and black spots on the head (χ^2^ = 9.6442, p = 0.0018, N = 10). Head size was not associated with female choice (χ^2^ = 0.3278, P = 0.5669, N = 10).

### Experimental manipulation of male black pigment on the head

**Figure 6:**
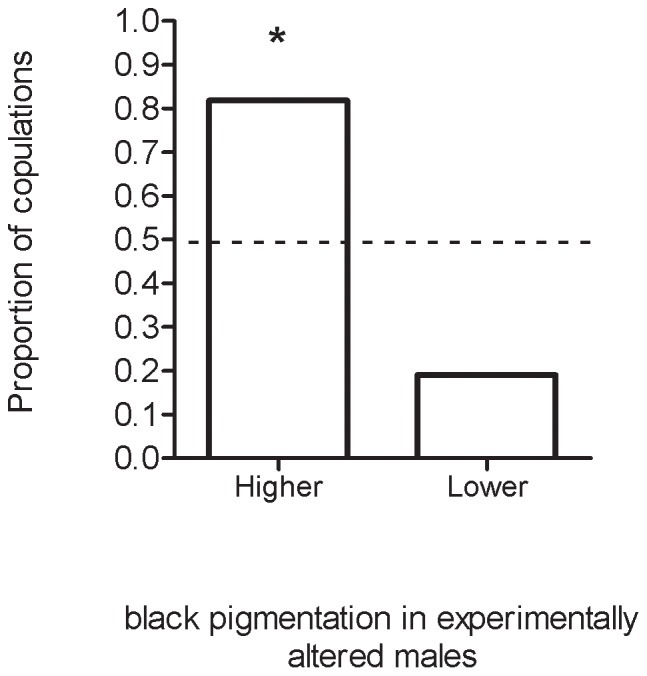
Proportion of copulations of males experimentally manipulated to have high and low black pigmentation on the head. * Represents statistical difference in proportions (see text). The dotted line represents the null expectation of 50%.

Copulations occurred in 11 out of 62 mating trials made with males whose proportion of black pigmentation on the head were previously manipulated. Males experimentally manipulated to have a higher proportion of black facial pigment were more chosen by the female as a sexual mate than males with apparent lower proportion (9 out of 11 males; Fisher's exact test: P = 0.01, N = 11; Fig. 6). White painting on the thorax did not affect female choice of sexual partners (Fisher's exact test: P = 1, N = 11).

The data table used for statistical analyses is available in figure S9.

## Discussion

In *P. simillimus*, the black facial pigmentation of males proved to be a sexually selected signal. This pigmentation is dimorphic, as it is larger and more variable in males than in females. Furthermore, female choice of sexual partner is linked to the proportion of black pigment in the male's head so that males with high rather than low proportion of black pigment on the head are chosen as sexual partners. Finally, males experimentally manipulated to have higher black pigmentation on the head were preferred by females as sexual partner compared to those with low pigmentation, suggesting that females evaluate male coloration. Thus, the proportion of black pigment on the head of males is a sexually selected signal used by females during the choice of the sexual partner. One important point was the low rate of successful copulations in our experiments. A recent work showed that males of *P. dominula* avoid copulate with workers [36]. Since in our experiments we did not discriminate among future foundresses and late workers it is possible that *P. simillimus* males present the same preference. Anyway, our analyses focused only in reproductively receptive females.

We found no evidence that the black pigmentation on the head is associated with the establishment of dominance relationships among males. During each dominance trial between males of *P. simillimus*, the frequency of darts and bites were low, and mounting was not observed. In contrast, males of other *Polistes* species often exhibit such aggressive behaviors [17], [18], [22]. The rarity in the frequency of aggressive behavior suggests that males of *P. simillimus* differ from other male *Polistes* studied so far. However, interpretation of these results should be considered with caution because of the lack of more complete information about the male behavior in this species.

The stronger negative correlation between the male proportion of black pigmented on the head and body size is quite surprising. In *Polistes*, it is known that quality signals are generally positively associated with body size [25], [26], [27] and individual identity signals are not [24]. Our findings suggest that the signal we found for *P. simillimus* have different properties compared to previous *Polistes* visual signals studied so far. Most importantly, despite the association with the proportion of vertex black pigmented, body size doesn't predict female choice or male-male dominance, so that these two visual traits may not convey the same information for females.

The yellow spots on the second abdominal segment in *P. simillimus* are sexually dimorphic and despite not associated with male-male dominance relations, they are associated with female choice of sexual partner. Since female choice was affected by the interaction between the proportion of vertex black and the SI, it is likely that females evaluate both yellow and black spots before choose a male. The yellow spots on the second abdominal segment of *P. dominula* males, probably homologous to that of *P. simillimus*, are sexually selected signals [22]. They are sexually dimorphic, and they are used to mediate both male-male dominance relations and female choice of sexual partners. Further experimental investigation will be necessary to clarify the function of *P. simillimus* abdominal spots.

As long as we can tell, both abdominal spots in *P. dominula* and the black spot on *P. simillimus* males share similar properties, as they are sexually dimorphic, visual, conspicuous signals, and have continuous variation. However, they differ in location and in the kind of pigment they are composed. In wasps, yellow spots are formed by pterines while dark spots are formed by melanin [37]. The independent evolved sexual signals in *Polistes* suggest that sexual selection may be common in these insects.

In many systems, sexual signals provide useful information to the receiver. For example, females of *P. dominula* whose sexual partner had more regular spots (preferred phenotype) survive longer than females whose sexual partner had less regular spots (not preferred phenotype) [38]. Thus, females' choice based on male's abdominal spots maximizes their direct fitness [38]. Future studies with *P. simillimus* will be interesting to verify if independently evolved sexual signals convey the same type of information or if there are other factors mediating the evolution of female choice in social wasps.

The natural history of *Polistes* suggests a favorable scenario for the evolution of female choice. In most species, females are genetically monogamous [20]. They copulate before winter, but only begin to fertilize the eggs in early spring. In addition, several generations of sterile workers are produced before future queens [15]. As a result, the sperm must be stored for several months, and thus, choosing good quality sexual partner may be important. The major challenge for the study of sexual selection in *Polistes* is observing sexual behavior in natural environments, essential for the interpretation of recent advances obtained from the laboratory experiments.

Visual signals in *Polistes* seem to be involved in communication at closer distances, as they are considered mediators of aggressive and/or sexual interactions [22], [25]–[27]. In both cases, the positioning of individuals face to face is common and thus communication is only effective if the visual acuity is appropriate. The compound eyes of insects have excellent spatial resolution at short distances [39]. In support, evidence suggests that wasps are able to perceive different characteristics of visual signals, such as patterns of disruption [25], irregularity of contour [22] and the area of the body spots [27]. Thus, it seems that the social wasp's vision is sufficiently accurate to detect the variability in body coloration typical of *Polistes*.

Most research on insect communication focuses on chemical signals [40]. For example, the role of cuticular hydrocarbons in communication among social insects is well known. These substances are used as signal of fertility, dominance and also for recognition of nest mates [40]–[44]. However, the visual communication can also be important. For example, in females of *Polistes fuscatus*, facial and abdominal marks are signals for individual recognition [24]. In females of *P. dominula*, *P. exclamans* and *P. satan*, facial markings are signals of quality that inform about the fighting ability of females [25], [26], [27]. Finally, in males of *P. dominula* and *P. simillimus*, visual signals are involved in intra and/or intersexual competition [22]. Together, this body of information suggests that the visual channel has been explored in different ways, highlighting the potential of social wasps as models for the study of visual communication in insects.

## Supporting Information

Figure S1 **Proportion of black pigment on a male head (A), determined as the black pigment area enclosed by the white line in B, with respect to the area enclosed by the white line in C.** Note the exclusion of the ocelli and antennal sockets area (grey circles).
(TIF)

Figure S2 **Spots of a male second abdominal tergite.** Dorsal (A) and lateral (B) views of the abdomen showing the spots (arrows). The spot shape index was calculated from the area of each spot (C) and the length (L) and height (H) of the largest sphere that can be inserted into each spot. In all images, the male anterior side is to the left.
(TIF)

Figure S3 **Measurement of maximum head width (white line), used to infer the body size.**
(TIF)

Figure S4 **Abdominal spots in males (A–D) and females (E–H).** Each image shows one of the two spots in each individual and tin all images the wasp's anterior side is to the left.
(TIF)

Figure S5 **Variation in the SI of males and females.** SI is higher in males than in females. Each circle represents one individual and the arrows indicate the mean. * Indicates statistical difference between the classes (see text).
(TIF)

Figure S6 **Relation between head size and SI.** Males have a stronger negative relation compared to females.
(TIF)

Figure S7 **Variation in head size of males and females.** Males are smaller than females. Each circle represents one individual and the arrows indicate the mean. * Indicates statistical difference between the classes (see text).
(TIF)

Figure S8 **SI of males chosen and not chosen by females as sexual partners.** Mean and standard deviation are presented. * Indicates statistical difference between the classes (see text).
(TIF)

Figure S9 **The data table used for statistical analyses.**
(XLSX)
