# Supplementary figures and images for: Sexy Faces in a Male Paper Wasp
Source: PLoS One. 2014 May 21;9(5):e98172. doi: 10.1371/journal.pone.0098172 (PMC4029984; doi:10.1371/journal.pone.0098172)

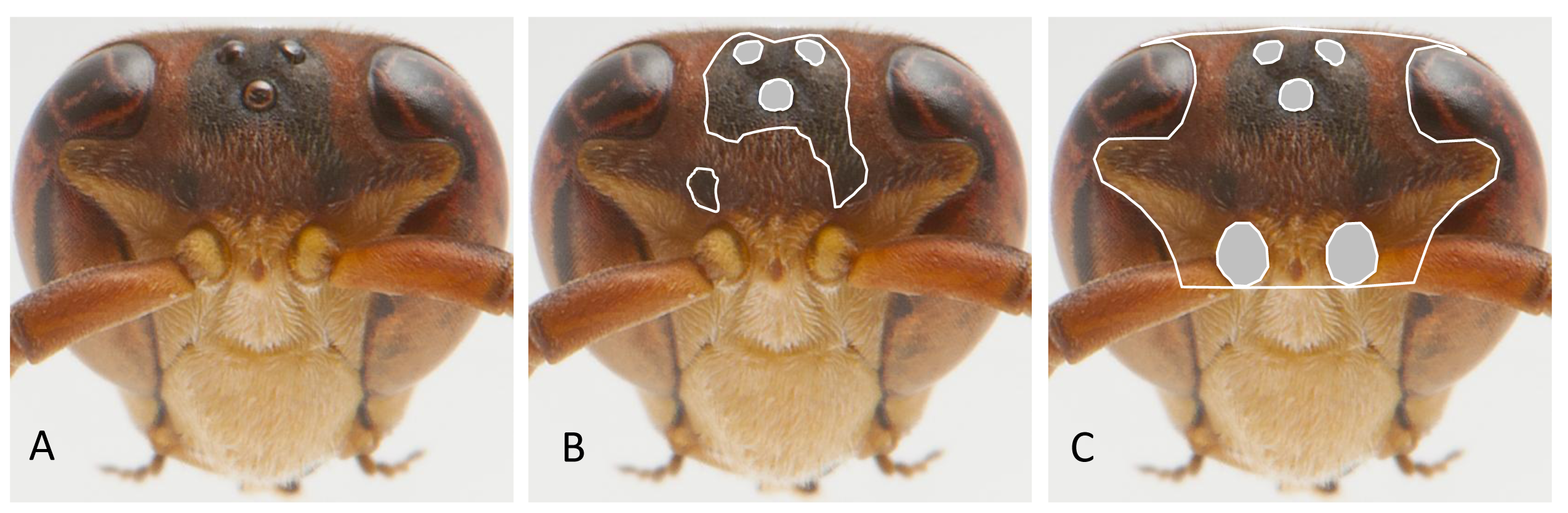

Supplement: Figure S1 — Proportion of black pigment on a male head (A), determined as the black pigment area enclosed by the white line in B, with respect to the area enclosed by the white line in C. Note the exclusion of the ocelli and antennal sockets area (grey circles). (TIF) [file pone.0098172.s001.tif]

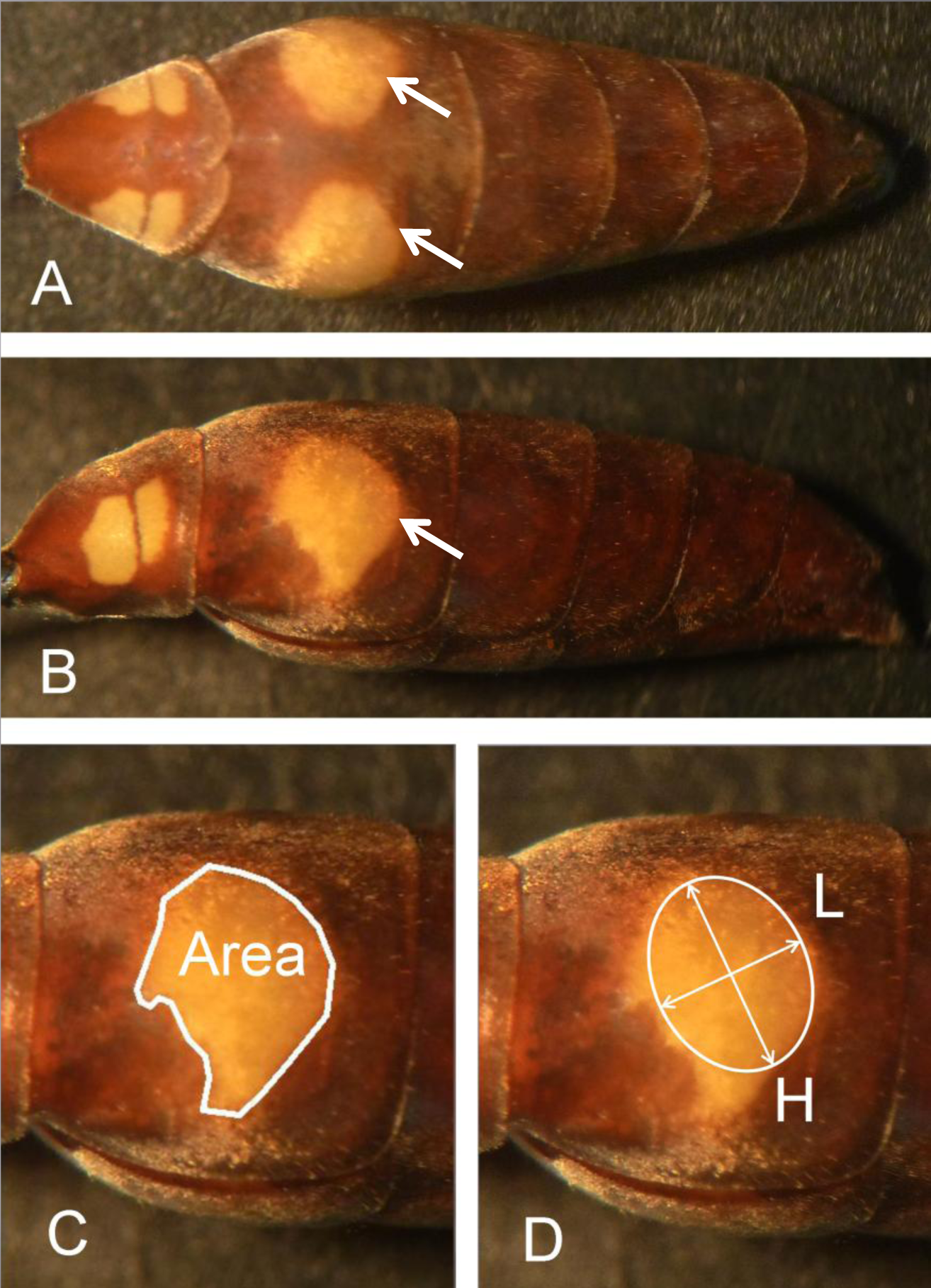

Supplement: Figure S2 — Spots of a male second abdominal tergite. Dorsal (A) and lateral (B) views of the abdomen showing the spots (arrows). The spot shape index was calculated from the area of each spot (C) and the length (L) and height (H) of the largest sphere that can be inserted into each spot. In all images, the male anterior side is to the left. (TIF) [file pone.0098172.s002.tif]

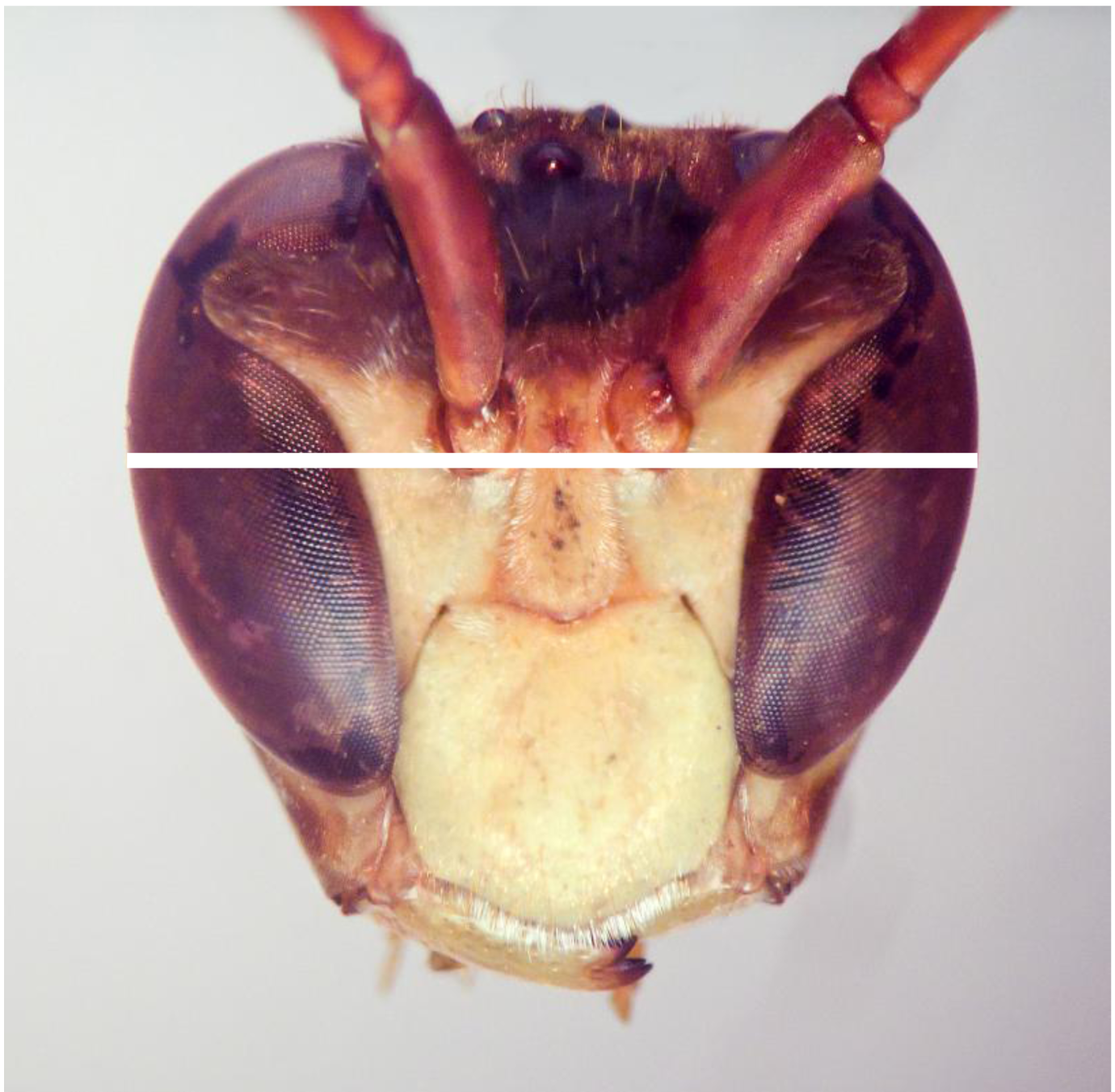

Supplement: Figure S3 — Measurement of maximum head width (white line), used to infer the body size. (TIF) [file pone.0098172.s003.tif]

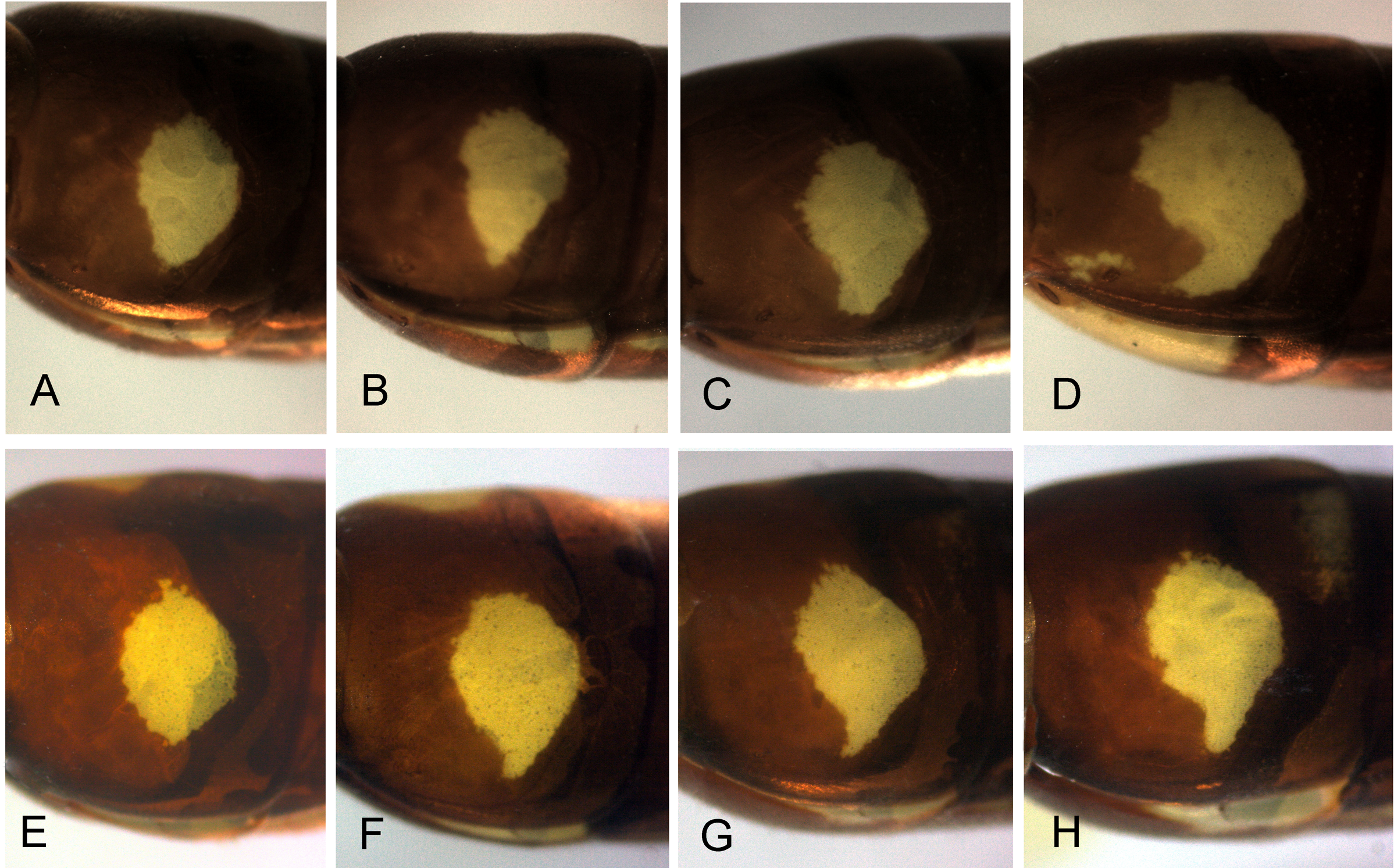

Supplement: Figure S4 — Abdominal spots in males (A–D) and females (E–H). Each image shows one of the two spots in each individual and tin all images the wasp's anterior side is to the left. (TIF) [file pone.0098172.s004.tif]

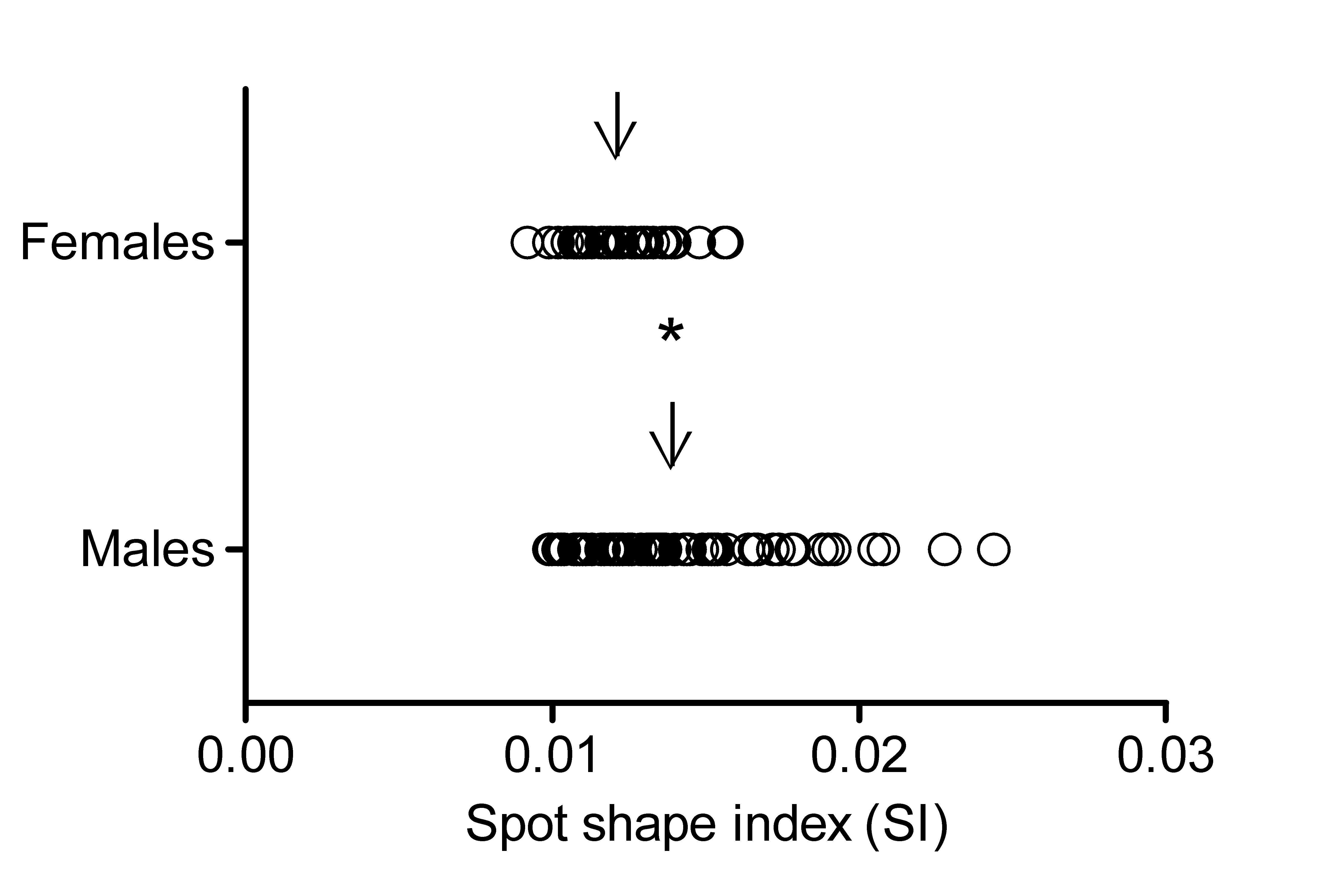

Supplement: Figure S5 — Variation in the SI of males and females. SI is higher in males than in females. Each circle represents one individual and the arrows indicate the mean. * Indicates statistical difference between the classes (see text). (TIF) [file pone.0098172.s005.tif]

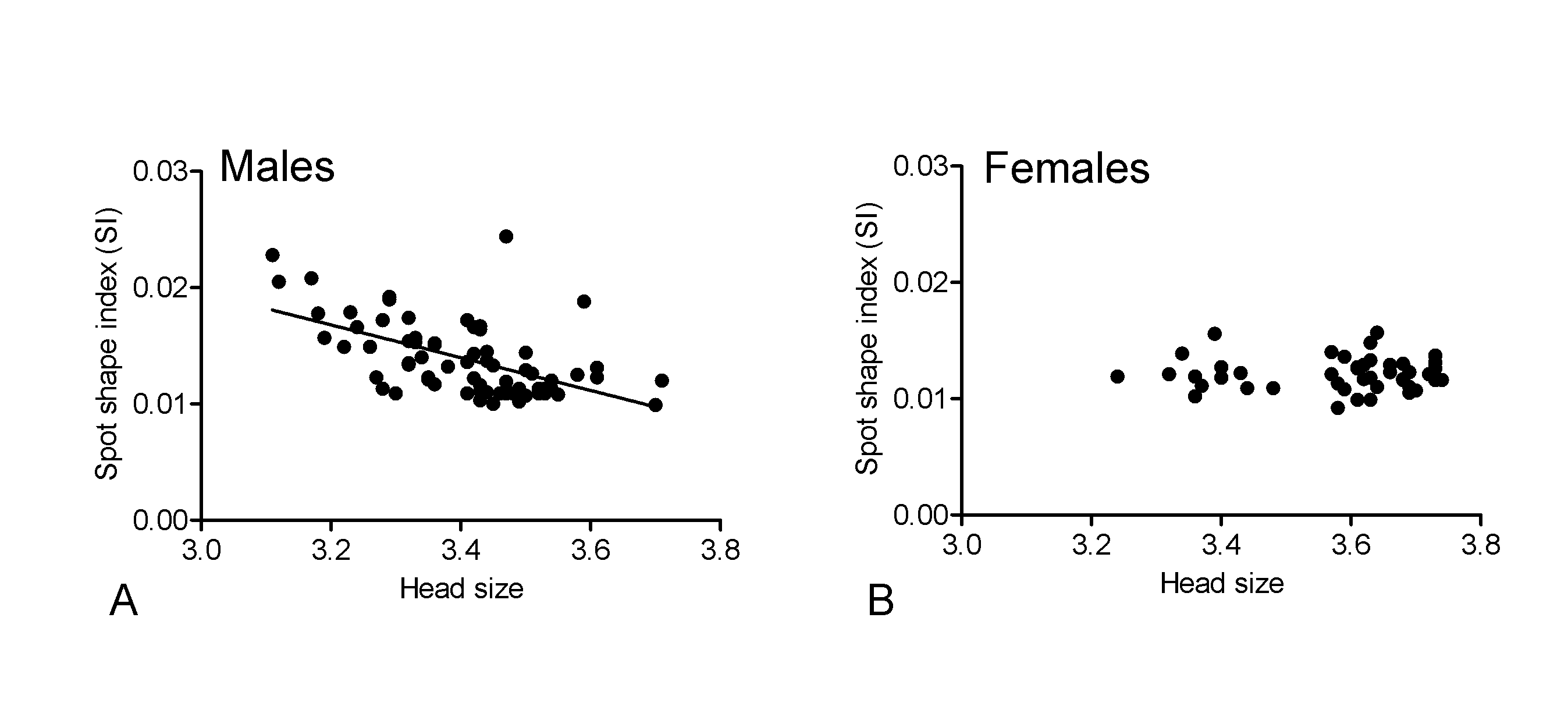

Supplement: Figure S6 — Relation between head size and SI. Males have a stronger negative relation compared to females. (TIF) [file pone.0098172.s006.tif]

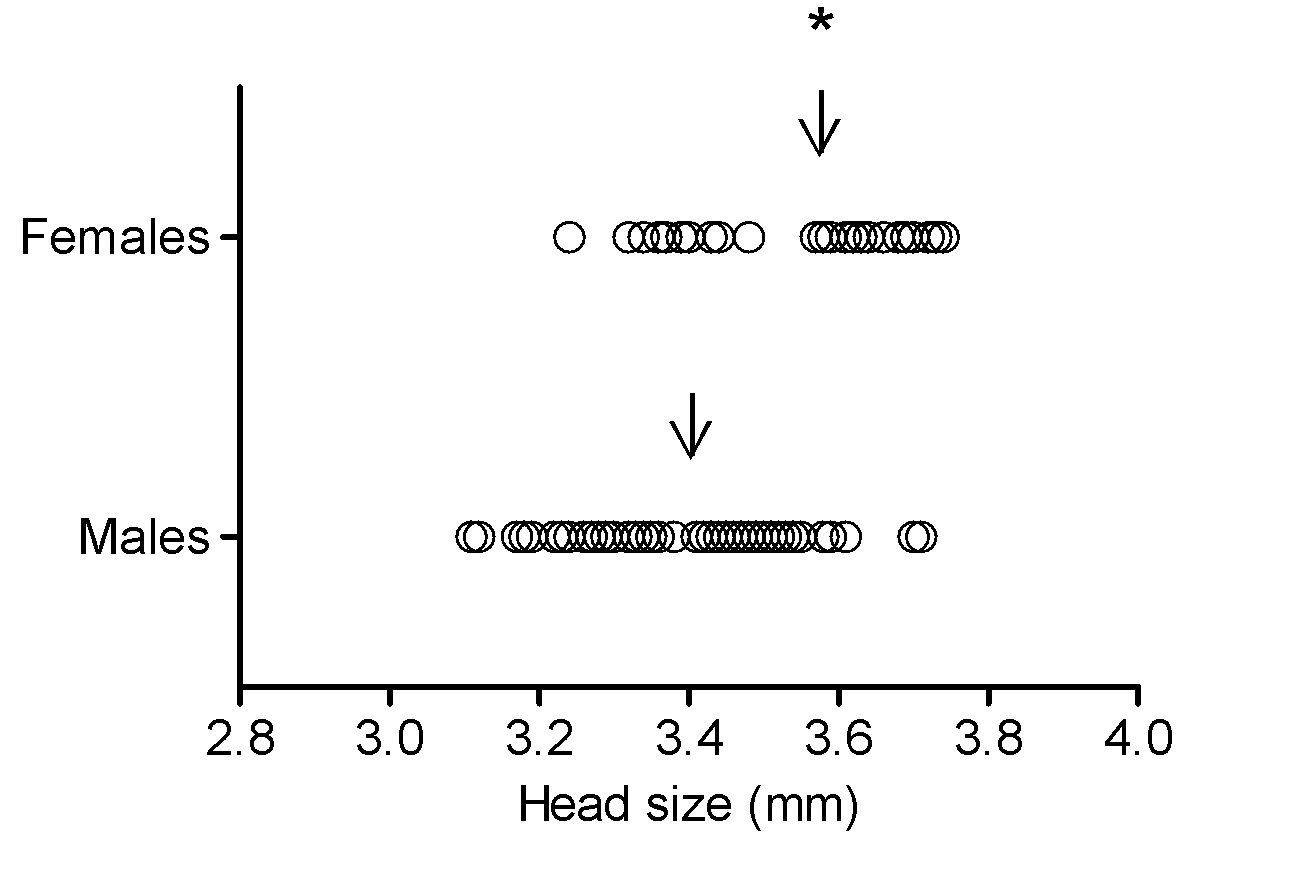

Supplement: Figure S7 — Variation in head size of males and females. Males are smaller than females. Each circle represents one individual and the arrows indicate the mean. * Indicates statistical difference between the classes (see text). (TIF) [file pone.0098172.s007.tif]

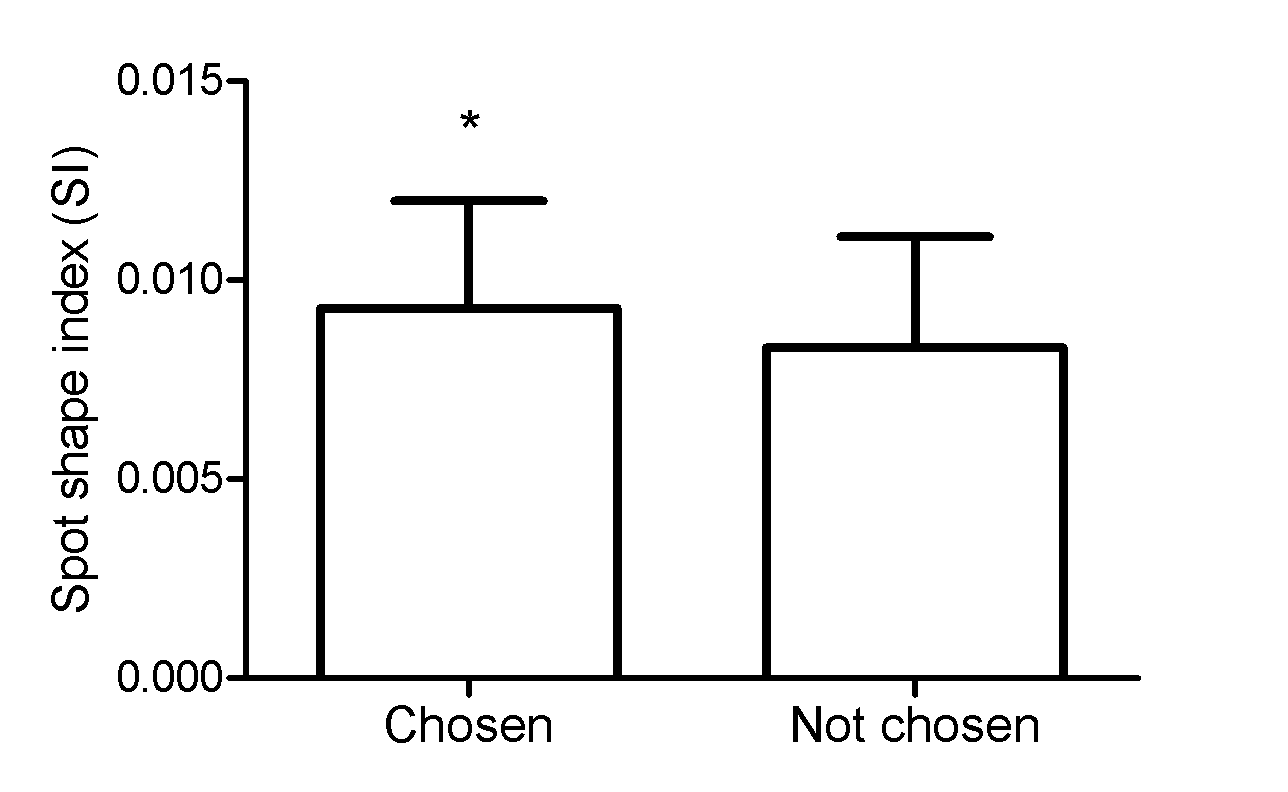

Supplement: Figure S8 — SI of males chosen and not chosen by females as sexual partners. Mean and standard deviation are presented. * Indicates statistical difference between the classes (see text). (TIF) [file pone.0098172.s008.tif]
